# Draft Genome Sequences of Two Unclassified *Chitinophagaceae* Bacteria, IBVUCB1 and IBVUCB2, Isolated from Environmental Samples

**DOI:** 10.1128/genomeA.00787-17

**Published:** 2017-08-17

**Authors:** Russell J. S. Orr, Stephane Rombauts, Yves Van de Peer, Kamran Shalchian-Tabrizi

**Affiliations:** a Section for Genetics and Evolutionary Biology (EVOGENE), Department of Biosciences, University of Oslo, Oslo, Norway; b Department of Plant Biotechnology and Bioinformatics, Ghent University, Ghent, Belgium; c VIB Center for Plant Systems Biology, Ghent, Belgium

## Abstract

We report here the draft genome sequences of two *Chitinophagaceae* bacteria, IBVUCB1 and IBVUCB2, assembled from metagenomes of surface samples from freshwater lakes. The genomes are >99% complete and may represent new genera within the *Chitinophagaceae* family, indicating a larger diversity than currently identified.

## GENOME ANNOUNCEMENT

Advances in high-throughput sequencing, coupled with decreasing costs, have increased the number of available bacterial genomes almost exponentially. Genome sequencing, however, has traditionally been limited to species that can be held and grown in culture due to the high DNA volumes needed. A predominant focus on cultivable species has led to a genome bias, and, as a result, true bacterial diversity is poorly represented. Metagenomic studies are rectifying this bias and have already revealed a large novel diversity (1). However, metagenomics remains limited, with many ecosystems yet to be sampled. We aim to expand species richness by identifying novel bacteria from varied environmental samples. Here, we present the draft genomes of two unclassified *Chitinophagaceae* bacteria, which were surface-isolated from freshwater lakes in Norway (Årungen, Ås) and Japan (Tsukuba, Ibaraki).

DNA was isolated using the standard phenol-chloroform protocol with ethanol precipitation and subsequent cleaning using Zymo genomic cleaner and concentrator. DNA was prepared and sequenced on an Illumina HiSeq 2500 (150-bp paired-end reads; 350-bp insert size) and PacBio RS2 with P6-C4 chemistry (20 kb) at the Norwegian Sequencing Centre. Metagenome drafts were assembled using SPAdes version 3.9.0 (2); single genomes were separated with MetaBAT (3); and quality was assessed with CheckM (4). Separate genomes were scaffolded using LINKS (5), and gaps were closed with Sealer (6). Genome assemblies were evaluated with PROmer (7) and REAPER (8) before being improved with Pilon (9). Genomes were annotated using the NCBI Prokaryotic Genome Annotation Pipeline (10). Taxonomical rank was established upon evaluation of CheckM (4), PhyloSift (11), and a megaBLAST search against the NCBInr database.

*Chitinophagaceae* bacterium IBVUCB1 was assembled into two scaffolds constituting six contigs with a sequence length of 3.41 Mb and a GC content of 42.64%. Scaffold *N*_50_ was 1.92 Mb with an Illumina coverage of 143× and a PacBio coverage of 9×. CheckM estimated genome completeness at 99.01% with no contamination or strain heterogeneity. The genome constitutes 3,056 genes, 43 RNAs, 36 tRNAs, 3 noncoding RNAs (ncRNAs), and 5 pseudogenes.

*Chitinophagaceae* bacterium IBVUCB2 was assembled into three scaffolds constituting four contigs with a total sequence length of 3.99 Mb and a GC content of 38.42%. The scaffold *N*_50_ was 2.26 Mb with an Illumina coverage of 32× and a PacBio coverage of 8×. CheckM estimated genome completeness at 99.51% with no contamination or strain heterogeneity. The genome constitutes 3,527 genes, 42 RNAs, 36 tRNAs, 3 ncRNAs, and 25 pseudogenes.

The genomes were confirmed as novel *Chitinophagaceae* bacteria according to a BLASTn search of 16S queries against the NCBInr database: IBVUCB1 had a 93% identity to *Sediminibacterium salmoneum* 16S (NR_044197), and IBVUCB2 had a 96% identity to the 16S of the same species. IBVUCB1 and IBVUCB2 had a 94% 16S identity to each other. The low identity to known *Chitinophagaceae* spp. may suggest IBVUCB1 and IBVUCB2 as new genera, indicating a larger diversity than currently identified.

### Accession number(s).

The draft genomes of *Chitinophagaceae* bacteria IBVUCB1 and IBVUCB2 sequenced under this project have been deposited at DDBJ/EMBL/GenBank under the accession numbers NFUW00000000 and NFUV00000000, respectively. These biosamples (SAMN06840505 and SAMN06840506, respectively) are part of BioProject PRJNA384425.
